# Appraisal on patient compliance and factors influencing the daily regimen of anti-tubercular drugs in Mangalore city: A cross-sectional study

**DOI:** 10.12688/f1000research.109006.2

**Published:** 2022-07-27

**Authors:** Rohith Motappa, Tuba Fathima, Himani Kotian

**Affiliations:** 1 Department of Community Medicine, Kasturba Medical College, Mangalore, Manipal Academy of Higher Education, Manipal, India; 2 Department of Community Medicine, Yenepoya Mecical College, Mangalore, Karntaka, 575003, UK

**Keywords:** Tuberculosis, Daily Regimen, Compliance

## Abstract

**Background:** Globally, India is the country with the highest tuberculosis (TB) burden with respect to the number of new cases occurring each year. Annual incident cases of TB in India accounts for more than 25% of total TB morbidity and mortality worldwide. Several factors have been associated with the adherence of TB medication, which can be broadly classified as patient/personal, social, structural and health service. The aims of the present study were to determine the compliance to daily regimen of directly observed treatment, short-course (DOTS) therapy among TB patients registered at the Tuberculosis Unit (TU) of Mangalore and to identify the factors influencing non-compliance for treatment.

**Methods:** A cross sectional study was performed. The study sample was drawn from the TUs, General Hospital, Mangalore, after taking permission from District TB Officer. The names and addresses of TB patients were collected from treatment cards. The TB patients were approached at their homes/DOTS centers/Primary Health Centre’s (PHCs) with the help of senior treatment supervisors.

**Results:** It was found that patients positive for human immunodeficiency virus (HIV) were more likely to be non-adherent, which was statistically significant. Amongst the participants, 66 (33%) were diabetic and 28.8% of them were found to be non-adherent. The proportion of non-adherence was 27 times higher in those with poor patient provider relationships. Patients who reported to have side effects of TB medication were 5.23 times more likely to be non-adherent.

**Conclusions:** Advice on routine consultation with the health care facility, adherence to treatment regimen and education about its benefits should be the prime focus of providing health education to all TB patients, both at the individual and community levels.

## Introduction

Tuberculosis (TB) is a rampant infectious disease caused by the bacteria, *Mycobacterium tuberculosis.* It is one of the most important socioeconomic global diseases with a high mortality rate.

Global estimation shows around 10.0 million (range, 8.9–11.0 million) people fell ill with TB in 2019, a number that has also been declining very slowly in recent years according to the Global tuberculosis report 2020 by World Health Organization (WHO) (Accessed on 28-11-2021).

Globally TB is the 13th leading cause of death and the second leading infectious killer after COVID-19 (above human immunodeficiency virus (HIV)/acquired immune deficiency syndrome (AIDS)) according to WHO (Accessed on 28-11-2021). India accounts for more than one-fourth of total TB cases and deaths worldwide. A total of 2.8 million new cases and 470,000 deaths due to TB, were reported in 2018, according to WHO global report. ^1^^,^^2^

National and international efforts over the last few decades regarding the prevention of TB, and its early diagnosis and treatment have been guided by the directly observed treatment, short-course (DOTS) strategy and followed by the Stop TB strategy. Recently, the WHO End TB strategy highlighted global targets, including: i) To decrease TB deaths by 90%; ii) to reduce incident cases by 80% by 2030 and iii) to ensure that there is no family burden due to TB costs. ^1^ To individuals diagnosed with TB, the DOTS strategy involves the delivery of a standard short course of drugs lasting six months for new patients and eight months for retreatment patients. ^3^

The Supreme Court of India in February 2017 directed that all new patients should be administered a daily regimen of TB drugs. Under the new daily drug regimen, TB patients will be given fixed-dose combinations, three or four drugs in specific dosages in a single pill, on a daily basis. The patient under the new regimen will have a reduced pill burden, as instead of seven tablets, patients need to consume only two or three tablets, according to their weight band. ^4^^,^^5^

Daily regimens recommended by WHO have been shown to be highly effective for both preventing and treating TB, however poor adherence to anti-TB medication is a major barrier in its global control. Nearly 20-50% of patients fail to complete therapy and as TB is a rampant communicable disease, this leads to prolonged infectiousness, drug resistance, relapse, and death. ^6^^,^^7^

Several factors have been associated with the adherence of TB medication, which can be broadly classified as social, structural, health service and patient/personal. ^5^

TB is mainly a disease of the poor in a country like India. Low income and poor living conditions, including overcrowding, contribute to the economic and structural factors. ^4^

TB can also interfere with the mental health of an individual. Many studies report a high prevalence of psychiatric issues among patients with drug-resistant TB, which significantly correlates with severity and duration of the disease and can also be associated with poorer adherence to anti-TB treatment. ^1^^,^^8^^–^^10^

Stigmatization has elicited a lot of self-denial among TB patients, hence the non-adherence. Families with low literacy level believe that merely associating with TB patients may cause them to have the disease and may create differences of opinion in the household about providing support to the patients, thereby leading to non-adherence. Also, discrimination on the basis of the disease at healthcare facilities may sometimes exacerbate the problem with adherence to drug-taking behavior. ^11^

Non-compliance to therapy leads to resurgence and persistence of TB and is regarded as an important cause of the development of multidrug-resistant (MDR)-TB and relapse. Hence, this study focused on compliance rates of anti-TB regimen and to determine factors associated with eventual non-compliance ( Global tuberculosis report 2020). The aims and objectives of this study were to determine the compliance of daily regimen of DOTS therapy among TB patients registered at the Tuberculosis Unit (TU) of Mangalore, and to identify the factors influencing non-compliance for the treatment among the same group of patients.

## Methods

### Study design and ethical considerations

This was a **c**ross-sectional study that took place from 15 ^th^ July 2021 to 15 ^th^ September 2021.

The study was approved by the Institutional Review Board of Yenepoya Ethics Centre 2 (Protocol number-YEC2/770) and performed in accordance with the principles of the Declaration of Helsinki. Written informed consent was obtained from participants.

### Study population

Multiple TUs attached to the Designated Microscopy Centre in Government Hospital located in Mangalore were selected. This study was undertaken in those TUs. TB cards were used to approach the TB patients.

### Study site

Selected Primary Health Centre’s (PHCs) of Mangalore city and Wenlock district hospital in Dakshina Kannada district.

### Sampling

With the ongoing pandemic of covid-19, convenient sampling was done to select the multiple tuberculosis units in Mangalore. All the TB patients registered in those Tuberculosis Units were included in the study. TB cards were used to approach those patients. The TB patients were approached at their homes/DOTS centers/PHCs with the help of senior treatment supervisors.

From previous studies, it was found that the compliance rate among TB patients is 50%. Considering 5% level of significance and 7% absolute precision around the specified rate, the minimum sample size recommended for the present study was calculated by the following formula:

$$n=\frac{Z\;{\left(1-\alpha /2\right)}^{2}*P*\left(1-P\right)}{d^{2}}={\left(1.96\right)}^{2}\times 0.50\times 0.050/{\left(0.07\right)}^{2}=196=200^{*}$$

*(To round off, this study only included 200 participants)

### Inclusion criteria

1. The sputum positive pulmonary TB patients (18 years and above) registered in three TU, who were on treatment.
2. Patients who had been on anti-TB drugs for at least three weeks of treatment.

### Exclusion criteria

1. Patients who were critically ill.
2. Pregnant and lactating women.
3. If the patient couldn’t be found after two consecutive home visits.

### Methodology

The study sample was drawn from the TU, General Hospital, Mangalore, after taking permission from the District TB Officer. The names and addresses of sputum-positive TB patients were collected from treatment cards. The TB patients were approached at their homes/DOTS centers/PHCs with the help of senior treatment supervisors. The study purpose was explained participants at the time of the interview. They were informed that, their participation in the study was voluntary and that they could withdraw from the study at any point of time. Maintenance of confidentiality about data and findings was assured to the participants and their consent was obtained. ^6^

### Study tools

A semi-structured, pretested questionnaire was developed and administered to individuals who were on treatment, with modifications relevant to local conditions. ^12^ The pilot study was performed on 20 people for pretesting. Statistical validation for the questionnaire was done by Cronbach’s Alpha (<0.7).

The questionnaire consisted of three parts:

i) Socio-demographic variables.
ii) Knowledge and attitude factors leading to non-adherence with anti-TB treatment (WHO Global tuberculosis report 2019; Accessed on 28-11-2021).
iii) Factors contributing to non-adherence of anti-TB treatment.

### Statistical analysis

Data were entered in Microsoft Excel 2016 (Microsoft Excel, RRID:SCR_016137) and analyzed using SPSS version 25 (IBM SPSS Statistics, RRID:SCR_019096). Data were interpreted in proportions and percentages, and Chi-square and P-values were obtained. Odds ratio values were obtained, and logistic regression analysis was performed to determine the likelihood of the factors involved in DOTS compliance in TB patients.

### Operational definitions

**Adherence** - Patients who not had missed even a single prescribed dose of TB drug were deemed as Adherent 

**Non–Adherence** - Patients who had missed at least one prescribed dose of TB drug were deemed as Non-Adherence

**Patient-Provider Relationship** - For differentiating between Good Patient-Provider relationships and Poor Patient-Provider relationships, we used 3 key traits of Health workers toward the patients, namely:

• Empathy towards the patients
• Effective communication with the patients and
• Shared decision-making regarding testing, treatment, and follow-up.

If all the 3 traits were deemed as satisfied by patients, they were categorized as Good Patient-Provider relationships, and even if one trait were not up to the mark according to the patients, it was categorized as Poor Patient-Provider relationships

## Results

Table 1 represents the socio-demographic details of the study participants. ^13^ Out of the 200 participants included in our study, 118 (59%) were men and 82 (41%) were women. Amongst the male and female participants, 30.5% and 32.9% were found to be non-adherent, respectively, and when compared regarding compliance, the difference was not statistically significant.

**Table 1.** Socio-demographic factors.

Variables		Adherent	Non-adherent	Chi-square	P-value
		N (%)	N (%)		
Sex	Male	82(69.5%)	36(30.5%)	0.131 ^a^	.758
	Female	55(67.1%)	27(32.9%)		
Age, years	18-28	24(61.5%)	15(38.5%)	3.805 ^a^	.283
	29-38	26(81.3%)	6(18.8%)		
	39-48	26(63.4%)	15(36.6%)		
	≥49	61(69.3%)	27(30.7%)		
Marital status	Single	33(73.3%)	12(26.7%)	.679 ^a^	.712
	Married	93(67.4%)	45(32.6%)		
	Widowed	11(64.7%)	6(35.3%)		
Religion	Hinduism	100(68%)	47(32%)	.206 ^a^	.902
	Islam	21(67.7%)	10(32.3%)		
	Christianity	16(72.7%)	6(27.3%)		
Education	No education	9(52.9%)	8(47.1%)	15.885 ^a^	.003
	Elementary school	7(50.0%)	7(50.0%)		
	Middle school	41(61.2%)	26(38.8%)		
	High school	49(71.0%)	20(29.0%)		
	University	31(93.9%)	2(6.1%)		
Type of residence	Urban	86(66.2%)	44(33.8%)	0.948 ^a^	.330
	Rural	51(72.9%)	19(27.1%)		
Income per month	Less than ₹5000	88(65.2%)	47(34.8%)	2.115 ^a^	.193
	More than ₹5000	49(75.4%)	16(24.6%)		
Type of family	Nuclear	95(70.9%)	39(29.1%)	1.327 ^a^	.515
	Joint	27(65.9%)	14(34.1%)		
	Three generation	15(60%)	10(40%)		
Occupation	Government servant	3(100%)	0(0%)	11.928 ^a^	.064
	Own business	6(75%)	2(25%)		
	Laborer/Farmer	10(71.4%)	4(28.6%)		
	Housewife	30(63.8%)	17(36.2%)		
	Unemployed	27(52.9%)	24(47.1%)		
	Retired	10(76.9%)	3(23.1%)		
	Skilled workers	51(79.7%)	13(20.3%)		
Type of TB	Category 1	124(69.7%)	54(30.3%)	1.014 ^a^	.336
	Category 2	13(59.1%)	9(40.9%)		
Mode of therapy	Non-resistant TB- Oral only	114(69.5%)	50(30.5%)	.433 ^a^	.554
	MDR TB- Oral or iv	23(63.9%)	13(31.5%)		
Site of disease	Pulmonary	91(68.9%)	41(31.1%)	0.002 ^a^	.968
	Extra pulmonary	46(68.7%)	21(31.3%)		
If extra pulmonary, site of TB	Abdomen	5(71.4%)	2(28.6%)	6.493 ^a^	.889
	Bone	2(100%)	0(0%)		
	Breast	2(100%)	0(0%)		
	Ear	1(100%)	0(0%)		
	Intestine	1(100%)	0(0%)		
	Lymph node	14(60.9%)	9(39.1%)		
	Meninges	2(100%)	0(0%)		
	Miliary	1(50%)	1(50%)		
	Pericardium	1(100%)	0(0%)		
	Pleural effusion	8(57.1%)	6(42.9%)		
	Skin	1(100%)	0(%)		
	Spine	8(72.7%)	3(27.3%)		
Distance from home to clinic, km	<3	52(70.3%)	22(29.7%)	.178 ^a^	.915
	3-5	38(67.9%)	18(32.1%)		
	>5	47(67.1%)	23(32.9%)		
Means of transportation	Foot	11(57.9%)	8(42.1%)	4.918 ^a^	.086
	Personal vehicle	75(75.8%)	24(24.2%)		
	Public transport	51(62.2%)	31(37.8%)		
Travelling time	Less than 30 minutes	116(69.5%)	51(30.5%)	.433 ^a^	.541
	More than 30 minutes	21(63.6%)	12(36.4%)		

^a^ significant values; TB, tuberculosis; MDR, multidrug-resistant.

A total of 39 (19.5%) of the participants belonged to the 28-25 age group, whereas the majority of the population were above the age of 49. Amongst them, 38.5% of the participants who were 18-25 years old and 30.7% who were over the age of 49 were non-adherent and this was found to be statistically insignificant.

This study showed that 135 (67.5%) of the participants had a monthly income of less than 5000 rupees and 65 (32.5%) earned more than 5000 rupees per month, of which 34.8% of the participants with income less than 5000 and 24.6% of those with income more than 5000 were found to be non-adherent. When they were compared with adherence, this difference was not statistically significant.

A total of 67 (33.5%) of the patients received education up to middle school and 69 (34.5%) of them had high school education. Almost 8% of the study participants received no education and 47% of these participants were non-adherent. When the educational status of the patients was compared with that of the compliance, the difference was found to be statistically significant (p=0.003).

Our study showed that 147 (73.5%) of the participants followed Hinduism, 31 (15.5%) followed Islam and 22 (11%) followed Christianity. Of the patients following Hinduism, Islam and Christianity, 32%, 32.3% and 27.3% were found to be non-adherent, respectively. In comparison with the compliance of these patients, the difference was not statistically significant.

A total of 134 (67%) of the patients belonged to a nuclear family and 12.5% lived in a three-generation family. A total of 29.1% of the patients belonging to a nuclear family and 40% of those living in a three-generation family were found to be non-adherent and when compared regarding compliance, the difference was not statistically significant.

Amongst our study participants, 51 (25%) were unemployed, 47 (23.5%) were housewives and 64 (32%) were reported to be skilled workers. A total of 47.1% of the unemployed, 36.2% of the housewives and 20.3% of the skilled workers were non-adherent to the TB regimen. In comparison with the compliance, there was no statistical significance.

A total of 178 (89%) of the patients belonged to category 1 and the remaining 11% were category 2 patients. A total of 30.3% of patients in category 1 and 40.9% of patients in category 2 were non-adherent. There was no statistical significance when these were compared with the compliance. A total of 18% of the participants suffered from MDR TB, of which 31.5% were non-adherent.

The site of TB was pulmonary in 132 (66%) of the patients and extra pulmonary in 22 (11%) of them. It was found that 31.1% of the patients with pulmonary TB and 31.3% suffering from extra pulmonary TB were non-adherent. The difference was statistically insignificant regarding the compliance.

Table 2 represents knowledge and attitude factors contributing to the poor compliance of anti-TB treatment. A total of 80 (40%) participants knew the correct causes of TB and 120 (60%) did not know the correct cause. Amongst them, 15% of the participants who knew the correct causes and 43.5% of them who did not know were found to be non-adherent. When compared in regards to the compliance, the difference was found to be statistically significant (p<0.001).

**Table 2.** Knowledge and attitude factors contributing to poor compliance with anti-TB treatment.

Variable		Adherent	Non-adherent	Chi-square	P-value
		N (%)	N (%)		
Causes of TB	Correct	68(85.0%)	12(15.0%)	16.823 ^a^	<0.001
	Incorrect	69(57.5%)	51(42.5%)		
Spread of TB	Correct	103(79.2%)	27(20.8%)	19.821 ^a^	<0.001
	Incorrect	34(48.6%)	36(51.4%)		
Prevention of TB	Correct	100(78.1%)	28(21.9%)	15.265 ^a^	<0.001
	Incorrect	37(51.4%)	35(48.6%)		
Curable	Correct	122(82.4%)	26(17.6%)	51.208 ^a^	<0.001
	Incorrect	15(28.8%)	37(71.2%)		
Seeking treatment	Hospital	128(68.1%)	60(31.9%)	.250 ^a^	.756
	General practitioner	9(75%)	3(25%)		
Knowledge about the names of TB drugs	Correct	33(82.5%)	7(17.5%)	4.542 ^a^	.037
	Incorrect	104(65.0%)	56(35.0%)		
Knowledge about the daily dosages of TB drugs	Correct	131(68.2%)	61(31.8%)	.163 ^a^	.686
	Incorrect	6(75%)	2(25%)		
Knowledge about the colors of TB drugs	Correct	121(76.6%)	37(23.4%)	22.777 ^a^	<0.001
	Incorrect	16(38.1%)	26(61.9%)		
Knowledge about the side effects of TB drugs	Correct	56(78.9%)	15(21.1%)	5.489 ^a^	.026
	Incorrect	81(62.8%)	48(37.2%)		
Awareness of daily regimen	Yes	123(78.8%)	33(21.2%)	35.177 ^a^	<0.001
	No	14(31.8%)	30(68.2%)		

^a^ significant values; TB, tuberculosis.

A total of 130 (65%) of the patients knew the exact mechanisms of the spread of TB and 70 (35%) of them did not know the correct mechanism. Of the people who knew the exact mechanism of spread, 20.8% were found to be non-adherent and 51.4% of the patients who did not know the mechanism were non-adherent. A total of 36% of the patients knew how to prevent TB. However, 64.5% of the study participants had no knowledge about side effects of TB drugs. These values were found to be statistically significant (p<0.001).

Participants who knew the names of the TB drugs were 40 (2%), while 160 (80%) did not know the names. Amongst them, 17.5% of the participants who knew the exact names and 35% of the ones who did not know the names were non-adherent. When this was compared with the compliance, the difference was found to be statistically significant (p=0.037).

A total of 156 (78%) participants were aware of the daily regimen and 22% had no awareness. A total of 21.2% of the participants who were aware of the daily regimen and 68.2% of those who were not aware, were found to be non-adherent. In regard to the compliance, these data were found to be statistically significant. Informants reported that the medication for the patients was given by the family members or treatment facilitators (health workers). The reason for being unaware of the regimen was found to be illiteracy.

As presented in Table 3, it was found that 4% of the study population who were HIV positive were non-adherent, which was statistically significant (p=0.002).

**Table 3.** Factors contributing to non-adherence to anti-TB treatment.

Variables		Adherent	Non-adherent	Chi-square	P-value
		N (%)	N (%)		
HIV status	Seronegative	135(71.1%)	55(28.9%)	11.475 ^a^	.002
	Seropositive	2(20.0%)	8(80.0%)		
Diabetes Status	Diabetic	47(71.2%)	19(28.8%)	.336 ^a^	.629
	Non-diabetic	90(67.2%)	44(32.8%)		
Smoking	Yes	19(67.9%)	9(32.1%)	.006 ^a^	1.000
	No	118(68.6%)	54(31.4%)		
Alcohol intake	Yes	32(72.7%)	12(27.3%)	.467 ^a^	.583
	No	105(67.3%)	51(32.7%)		
Relationship with patient provider	Good patient provider relationship	135(75.0%)	45(25.0%)	35.245 ^a^	<0.001
	Poor patient provider relationship	2(10.0%)	18(90.0%)		
Ran out of drugs at home	Yes	3(27.3%)	8(72.7%)	9.169 ^a^	.005
	No	134(70.9%)	55(29.1%)		
Regular supply of drugs	Yes	128(70.7%)	53(29.3%)	4.345 ^a^	.066
	No	9(47.4%)	10(52.6%)		
Side effects of TB medication	Yes	42(48.8%)	44(51.2%)	27.034 ^a^	<0.001
	No	95(83.3%)	19(16.7%)		
TB status disclosure to family	Yes	136(69.4%)	60(30.6%)	3.579 ^a^	.093
	No	1(25%)	3(75%)		
Support of family during treatment duration	Yes	136(69.7%)	59(30.3%)	5.590 ^a^	.035
	No	1(20.0%)	4(80.0%)		
Losing wages	Yes	51(63%)	30(37%)	1.934 ^a^	.215
	No	86(72.3%)	33(27.7%)		
Lack of money for transportation	Yes	4(22.2%)	14(77.8%)	19.632 ^a^	<0.001
	No	133(73.1%)	49(26.9%)		
Change of residence while on anti-TB medication	Yes	18(62.1%)	11(37.9%)	.650 ^a^	.517
	No	119(72.8%)	52(27.2%)		
Administration of TB drugs in presence of a family member or HCW	Yes	62(63.9%)	35(36.1%)	1.833 ^a^	.223
	No	75(72.8%)	28(27.2%)		
Treatment satisfaction at the health center	Yes	128(74.4%)	44(25.6%)	19.945 ^a^	<0.001
	No	9(32.1%)	19(67.9%)		
Interruption of TB treatment after three months	Yes	5(38.5%)	8(61.5%)	5.814 ^a^	.027
	No	132(70.6%)	55(29.4%)		
Boredom and isolation	Yes	12(48.0%)	13(52.0%)	5.565 ^a^	.023
	No	125(71.4%)	50(28.6%)		
Bad hospital setting	Yes	2(100%)	0(0%)	.929 ^a^	.335
	No	135(68.2%)	63(31.8%)		

^a^ significant values; TB, tuberculosis; HIV, human immunodeficiency virus; HCW, healthcare worker.

Amongst the participants, 66 (33%) were diabetic and 28.8% of them were found to be non-adherent. A total of 31.4% of the study population who had reported a history of smoking and 32.7% of the study participants who had reported alcohol intake gave a history of non-adherence. None of the variables in comparison with the compliance, showed any statistical significance.

A total of 180 (90%) of the patients reported to have a good patient provider relationship. Only 25% of them were found to be non-adherent. But 90% of the patients with a poor patient provider relationship were non-adherent and this difference regarding compliance was statistically significant (p<0.001).

A total of 91% of the patients reported no financial burden for transportation to the nearest hospital, which was also found out to be significant. A total of 43% of the study population experienced side effects of TB drugs, of which 51.2% were found to be non-adherent. This was found to be significant (p<0.001).

Regarding reasons for interruption to taking medication, variables such as feeling depressed, being sick, treatment course being long and the feeling that the drugs seem ineffective were found to be significant ( Table 4).

**Table 4.** Reasons for interruption of taking medications.

Variable		Adherent	Non-adherent	Chi-square	P-value
		N (%)	N (%)		
Feeling of depression and anxiety ever since disease was confirmed	Yes	39(50.6%)	38(49.4%)	18.489 ^a^	<0.001
	No	98(79.7%)	25(20.3%)		
Forgetting	Yes	1(8.3%)	11(91.7%)	21.417 ^a^	<0.001
	No	136(72.3%)	52(27.7%)		
Being busy with other work	Yes	1(16.7%)	5(83.3%)	7.702 ^a^	.013
	No	136(70.1%)	58(29.9%)		
Being out of home/town	Yes	1(16.7%)	5(83.3%)	7.702 ^a^	.013
	No	136(70.1%)	58(29.9%)		
Being sick	Yes	0(0.0%)	21(100.0%)	51.024 ^a^	<0.001
	No	137(76.5%)	42(23.5%)		
Treatment course is long	Yes	28(44.4%)	35(55.6%)	24.665 ^a^	<0.001
	No	109(79.6%)	28(20.4%)		
Fearing of pill burden	Yes	32(43.2%)	42(56.8%)	34.725 ^a^	<0.001
	No	105(83.3%)	21(31.5%)		
Treatment discontinued once symptoms resolve	Yes	0(0.0%)	6(100.0%)	13.451 ^a^	<0.001
	No	137(70.6%)	57(29.4%)		
Disease conditions have not been alleviated and the drugs seem ineffective	Yes	1(5.0%)	19(95.0%)	41.527 ^a^	<0.001
	No	136(75.6%)	44(24.4%)		
Appetite is influenced after taking drugs	Yes	34(47.9%)	37(52.1%)	21.675 ^a^	<0.001
	No	103(79.8%)	26(20.2%)		

^a^ significant values.

A total of 77 (38.5%) participants complained about being depressed and anxious since the disease was confirmed, of which 49.4% of them were found to be non-adherent, which was statistically significant (p<0.001).

It was found that 3% of patients cited being busy with other work, and among them 83.3% were non-adherent to TB medication. This was statistically significant (p=0.013).

A total of 63 (31.5%) of participants complained about the treatment course being long, among them 55.6% were found to be non-adherent. A total of 74 (37%) of the patients feared the pill burden and 56.8% of them were non-adherent. Both these were found to be statistically significant (p<0.001).

A logistic regression was applied to ascertain the effects of the aforementioned variables in the likelihood of the participants being non-compliant to the TB regimen ( Table 5). Non-adherence was 4.8 times higher in participants who did not know about the etiology of TB, 3.37 times higher in those who did not know the correct mechanism of the spread of TB, 11.57 times higher in those who believed that the disease was incurable, 2.5 times higher in patients who did not know the names of the TB drugs and 7.9 times higher in those who did not have an awareness of the daily regimen. ^8^

**Table 5.** Logistic regression detailing the adherence.

Variable		Adherent	Non-adherent	Adjusted odds ratio (95% CI)
		N (%)	N (%)	
Causes of TB	Correct	68(85.0%)	12(15.0%)	4.188 (4.188, 8.540)
	Incorrect	69(57.5%)	51(42.5%)	
Prevention of TB	Correct	100(78.1%)	28(21.9%)	3.378(1.810, 6.304)
	Incorrect	37(51.4%)	35(48.6%)	
Curable	Correct	122(82.4%)	26(17.6%)	11.574(5.554, 24.121)
	Incorrect	15(28.8%)	37(71.2%)	
Awareness of daily regimen	Yes	123(78.8%)	33(21.2%)	7.987(3.804, 16.768)
	No	14(31.8%)	30(68.2%)	
Patient provider relationship	Good patient provider relationship	135(75.0%)	45(25.0%)	27.000(6.029, 120.924)
	Poor patient provider relationship	2(10.0%)	18(90.0%)	
Side effects of TB medication	Yes	42(48.8%)	44(51.2%)	5.238(2.737, 10.025)
	No	95(83.3%)	19(16.7%)	
Support of family during treatment duration	Yes	136(69.7%)	59(30.3%)	9.220(1.009, 84.261)
	No	1(20.0%)	4(80.0%)	
Satisfied with treatment at the health center	Yes	128(74.4%)	44(25.6%)	6.141(2.589, 14.570)
	No	9(32.1%)	19(67.9%)	
HIV status	Seronegative	135(71.1%)	55(28.9%)	9.818(2.021, 47.709)
	Seropositive	2(20.0%)	8(80%)	
Feeling of depression and anxiety ever since disease was confirmed	Yes	39(50.6%)	38(49.4%)	3.819(2.041, 7.146)
	No	98(79.7%)	25(20.3%)	
Being busy with other work	Yes	1(16.7%)	5(83.3%)	11.724(1.340,102.573)
	No	136(70.1%)	58(29.9%)	
Treatment course is long	Yes	28(44.4%)	35(55.6%)	4.866(2.546, 9.299)
	No	109(79.6%)	28(20.4%)	
Fearing of pill burden	Yes	32(43.2%)	42(56.8%)	6.562(3.404, 12.653)
	No	105(83.3%)	21(31.5%)	

^a^ significant values; TB, tuberculosis; HIV, human immunodeficiency virus.

Non-adherence was found to be 27 times higher in patients with poor patient provider relationship, 5.23 times higher in patients who reported to have side effects of TB medication, 9.22 times higher in participants who did not receive support from their family during the treatment duration and 6.14 times higher in patients who weren't satisfied with the treatment at the health center ( WHO).

Non-adherence was 3.8 times higher in participants who complained about feeling depressed and anxious since the disease was confirmed, 11.72 times higher in patients who were busy with other work and 4.3 times higher inpatients whose appetite was influenced after taking TB medication. Participants who regarded the treatment course to be long and the ones who feared the pill burden were 4.8 and 6.5 times more likely to be non-adherent to the treatment, respectively ( WHO).

## Discussion

Overall adherence was not up to the mark, as expected. Due to the unprecedented pandemic of COVID-19, which played a significant role in the non-adherence habits of the study participants. ^14^

The present study showed that non-adherence was quite high in male participants who were predominantly unemployed. These findings were equivalent to a study done by Fang *et al*. ^15^

Patients who were from urban areas and had education until middle school were found to have greater percentages of non-adherence. These findings were similar to a study performed by Mekonnen and Azagew. ^16^

Knowledge about TB played a very important role in the rates of adherence. It was noted that a large proportion of the TB patients answered incorrectly about the aetiology of TB, modes of transmission and prevention methods, reflecting the poor basic knowledge about TB ( Global tuberculosis report 2020). A previous study conducted by Krasniqi *et al*. ^17^ showed a similar result.

Moreover, factors such as awareness about the long duration of the treatment and adverse effects of daily dosages, have played an important part in adherence to treatment ( Global tuberculosis report 2020).

HIV status was significantly associated with non-adherence, with 32% of HIV-positive patients being non-adherent. The reason for this could be attributed to the pill burden caused by antiretroviral therapy (ART) itself. These findings were concurrent with a previous study done by Naing *et al*. ^18^ The strategies to provide better anti-TB treatment to HIV-positive patients must be stressed upon in the programmes, since the number of HIV-positive patients who die due to TB is very high ( Global tuberculosis report 2019).

All HIV-positive patients should be administered drugs under the direct supervision of health authorities. Educating people about the association between TB and HIV is of great importance ( Global tuberculosis report 2019).

Many patients shared their view regarding the economic burden of TB treatment. Poverty plays an etiological role for TB disease and the cost of treatment amplifies economic hardships and reduces adherence. Studies performed by Mauch *et al*. ^19^ showed that cash incentives with food and transportation can improve treatment success rate. ^2^

Nearly 22% of the study participants who were experiencing side effects of the TB treatment were found to be non-adherent and it was significantly associated. These results indicated the importance of health education and counselling on the importance of continuing TB treatment until declared cured. Similar results were found in a study done by Kigozi *et al*. ^20^

A total of 31.5% of the participants expressed psychological stress after the diagnosis was made and many opined regarding the need for psychological support during treatment. ^8^ High prevalence of depression and anxiety in patients with TB contributing to the state of non-adherence is evident from other studies. ^17^ Psychosocial support improves treatment success rates and this in turn increases TB treatment adherence. TB diagnosis itself may be emotionally taxing on the patients. Collaboration of psycho-emotional care with TB treatment may help patients better face the challenges leading to non-adherence. ^21^

The analysis of TB compliance yielded a significant association with patients who considered the treatment course to be long, who deemed the drugs to be ineffective and who reported their appetite being reduced after anti-TB drugs. These variables demonstrate a link between major influencing factors for treatment adherence. Although, alcohol use, smoking, regular supply of drugs and distance from health care facility have been associated with high risk of treatment default as per the literature ( Global tuberculosis report 2020), these variables did not reflect the increased risk for TB treatment non-adherence in our study.

## Conclusions

In this qualitative assessment of non-adherence, it was revealed that treatment adherence was not satisfying. Intense health education and health awareness programs for TB patients, so they can be focused to adhere and motivate compliance towards TB treatment should be done at the individual and family level ( Global tuberculosis report 2020). Regular follow-up to health care centres and provision to information about the advantages and safety profile of drugs must be encouraged. Also, importance should be given to raise the satisfaction of patients with medical personnel and health services and establishing a regular monitoring system to identify patients with high risk for non-adherence should be initiated.

### Strengths of the study

• This study was done during the Covid-19 pandemic when all the resources and logistics were deviated towards curbing Covid-19 infection. With significant numbers indicating the non-adherence in our study, these study findings showcase that no matter which pandemic might arise, Tuberculosis cannot be brushed aside
• With all the States and UTs working hard toward eliminating TB by 2025, these study findings can provide an insight as to where we stand and how we can improve.

### Limitations

• As this study was done in Mangalore, these findings cannot be generalized for the Pan-India assumption
• This study considered the TB patients on the whole, without taking any details about the resistance patterns. Prevalence of non-adherence would have been even more comprehensible if we had taken the history of resistance towards any of the anti-tubercular drugs.

## Data availability

### Underlying data

Figshare: Data- Excel Sheet (Questionnaire results of the participants), https://doi.org/10.6084/m9.figshare.19113488 ^13^

### Extended data

Figshare: Questionnaire final (English).docx (Questionnaires used to collect the findings of the study), https://doi.org/10.6084/m9.figshare.19174745 ^12^

Data are available under the terms of the Creative Commons Attribution 4.0 International license (CC-BY 4.0).
