# Supplementary material for: Appraisal on patient compliance and factors influencing the daily regimen of anti-tubercular drugs in Mangalore city: A cross-sectional study
Source: F1000Res. 2022 Jul 27;11:462. Originally published 2022 Apr 26. [Version 2] doi: 10.12688/f1000research.109006.2 (PMC9296992; doi:10.12688/f1000research.109006.2)
Supplement: Peer review history

# Peer review: Reviewer response for version 2

**DOI:** 10.5256/f1000research.136645.r145609

**Published:** 2022-08-10

**Authors:** Poonam Naik

**Affiliations:** 1 Yenepoya Mecical College, Mangaluru, Karnataka, India

The modifications done by the author are accepted by me. 

 I have no further comments to make.

Is the work clearly and accurately presented and does it cite the current literature?

Yes

If applicable, is the statistical analysis and its interpretation appropriate?

Yes

Are all the source data underlying the results available to ensure full reproducibility?

Yes

Is the study design appropriate and is the work technically sound?

Yes

Are the conclusions drawn adequately supported by the results?

Yes

Are sufficient details of methods and analysis provided to allow replication by others?

Yes

Reviewer Expertise:

Operational Research, Tuberculosis, Geriatric health, Epidemiology, Public Health

I confirm that I have read this submission and believe that I have an appropriate level of expertise to confirm that it is of an acceptable scientific standard.

# Peer review: Reviewer response for version 1

**DOI:** 10.5256/f1000research.120456.r142668

**Published:** 2022-07-19

**Authors:** Ganesh Kumar Saya

**Affiliations:** 1 Department of Preventive and Social Medicine, Jawaharlal Institute of Postgraduate Medical Education and Research (JIPMER), Puducherry, Tamil Nadu, India

**Comments:**

1. In the results section of the abstract, please add more findings relevant to the study objectives.
2. The introduction may be further strengthened by adding studies carried out on TB- drug adherence during the COVID pandemic.
3. Conclusions should be based on the study findings. Specific recommendations based on the study findings may be included.
4. Do include the limitations of the study.

Is the work clearly and accurately presented and does it cite the current literature?

Yes

If applicable, is the statistical analysis and its interpretation appropriate?

Yes

Are all the source data underlying the results available to ensure full reproducibility?

Yes

Is the study design appropriate and is the work technically sound?

Yes

Are the conclusions drawn adequately supported by the results?

Partly

Are sufficient details of methods and analysis provided to allow replication by others?

Yes

Reviewer Expertise:

Maternal and child health, occupational health, spiritual health

I confirm that I have read this submission and believe that I have an appropriate level of expertise to confirm that it is of an acceptable scientific standard.

# Peer review: Reviewer response for version 1

**DOI:** 10.5256/f1000research.120456.r142666

**Published:** 2022-07-18

**Authors:** Aniwada Elias Chikee

**Affiliations:** 1 Department of Community Medicine, College of Medicine, University of Nigeria Teaching Hospital, Enugu, Nigeria

**TITLE**

- Is this compliance or adherence as all write up and tables were on adherence?
- Suggested title "Appraisal on patient adherence to and factors influencing the daily regimen of anti-tubercular drugs in Mangalore city: A cross-sectional study".

**ABSTRACT**

- Methods - include the study tool [Questionnaire], test statistics used [Chi square and Logistic regression], level of significance [p < 0.05].
- Result: It was found that non-adherence was more among; HIV positive (...%), Diabetics (28.8%), etc. Identified predictors of non- adherence were; having side effects 5.23 (confidence interval), etc.
- Conclusion: There was poor adherence among...Advice on routine...
- Key words: Remove "compliance" and use "adherence".

**MAIN BODY**

*Introduction*

- Remove all accessed to reference section after conclusion e.g., (accessed on 28-11-21)
- Remove Global tuberculosis report 2020 to reference section.
- The statement can read "according to WHO, global estimation shows..."
- Change 'Non-compliance' to 'Non-adherence' and 'compliance rate' to 'adherence rate'.
- Remove ( Global tuberculosis report 2020) and insert reference in superscript (all in last paragraph).

*Methods*

- Make Ethical consideration a subheading and include, "The study purpose was explained participants at the time of the interview. They were informed that, their participation in the study was voluntary and that they could withdraw from the study at any point of time. Maintenance of confidentiality about data and findings was assured to the participants and their consent was obtained." in the section.

*Study population *

- NB these were patients NOT YUs.
- Move Inclusion and exclusion criteria to this section.
Suggestion: 
  These were TB patients that accessed care during period of study. 
  Inclusion criteria
  1. The sputum positive pulmonary TB patients (18 years and above) registered in three TU, who were on treatment.
  2. Patients who had been on anti-TB drugs for at least three weeks of treatment.
  Exclusion criteria
  1. Patients who were critically ill.
  2. Pregnant and lactating women.
  3. If the patient couldn’t be found after two consecutive home visits.

*Study site*

- I would additionally state the nature of facilities e.g., Is this for TB alone or involved in other activities, days of TB activities, TB patient load/attendance. number and distribution of TUs in Mangalore.
- Change heading "sampling" to sample size.
- Why use 7% instead of 5% or 10%?
- The create separate heading for sampling.

*Sampling*

- NB - How were TB patients selected? E.g., simple random sampling, systematic, convenience, snowballing etc.
- The study sample was drawn from the TU, General Hospital, Mangalore, after taking permission from the District TB Office.

*Study tools before methodology*

- Remove "pilot study" and only use "pretesting" as pilot study was not done. These do not mean same thing.
- Remove accessed and WHO Global tuberculosis report.
- Change "Factors contributing to non-adherence of anti-TB treatment" to "reasons for non-adherence to anti-TB treatment".
  NB - Factors are derived by crosstabulation or bivariate and followed by multivariate analysis.
- Change "methodology" to "Data collection method".
- The names and addresses of sputum-positive TB patients were collected from treatment cards. The TB patients were approached at their homes/DOTS centers/PHCs with the help of senior treatment supervisors. They were interviewed using questionnaires.

*Statistical analysis*

- Chi-square was used for associations of characteristics of TB patients and adherence to treatment. Binary Logistic Regression was used to determine predictors/likelihood of adherence to TB treatment. Level of significance was at p < 0.05 

**RESULTS**

- Reduce reports tables. Report the findings and leave off its complements as they are seen in the table. E.g., highest age, highest income, education etc for adherent and non adherent.
- Table 1 change "Socio-demographic factors.' to "characteristics of TB patient based on adherence". Remove "a" in superscript for non significant values.
- Table 2 change "Knowledge and attitude factors contributing to poor compliance with anti-TB treatment" to "knowledge and attitude of TB patient based on adherence". Remove "a" in superscript for non significant values.
- Table 3 change "Factors contributing to non-adherence to anti-TB treatment" to" Reasons for non adherence to to anti-TB treatment"". Remove "a" in superscript for non significant values.
- Table 5 Change "Logistic regression detailing the adherence" to " knowledge and attitude factors predicting or influencing adherence to anti-TH treatment".
- NB - Remove columns with value and percentages. You do not use it at stage of multivariate analysis.
- Do similar analysis for characteristics of the patients - Title will be "Characteristics of patients predicting or influencing adherence to anti-TH treatment".
- NB; Always mention reference group in reporting regression, e.g., "Non-adherence was found to be 27 times higher in patients with poor patient provider relationship than those who do not".

**DISCUSSION**

- Remove all those citation in bracket and use superscript for corresponding number at reference section.
- Always state findings in studies you are making comparison for easy appreciation by readers. E.g., "These findings were equivalent to a study done by Fang *et al*. ^15 ^which reported that...".
- State reasons for your findings or why different from previous studies
- Include implication of some of your findings

**CONCLUSION**

- Remove qualitative assessment for you did quantitative study.
- Remove Global tuberculosis report 2020 . You do not reference in the conclusion.
- Be specific on areas, it is not satisfying.
- Recommendation should be based on findings.

Is the work clearly and accurately presented and does it cite the current literature?

Yes

If applicable, is the statistical analysis and its interpretation appropriate?

No

Are all the source data underlying the results available to ensure full reproducibility?

Yes

Is the study design appropriate and is the work technically sound?

Yes

Are the conclusions drawn adequately supported by the results?

No

Are sufficient details of methods and analysis provided to allow replication by others?

Yes

Reviewer Expertise:

Every area in medical science or Public Health with a bias on TB, HIV and substance use

I confirm that I have read this submission and believe that I have an appropriate level of expertise to confirm that it is of an acceptable scientific standard, however I have significant reservations, as outlined above.

# Peer review: Reviewer response for version 1

**DOI:** 10.5256/f1000research.120456.r135917

**Published:** 2022-06-06

**Authors:** Poonam Naik

**Affiliations:** 1 Yenepoya Mecical College, Mangaluru, Karnataka, India

The research study findings are important to understand the factors influencing compliance/ adherence to daily treatment regimes.

 The author may give more details on the following aspects:

1. Sampling method followed to select the sites and selection of study participants.
2. Operational definitions followed for certain important terminologies such as: adherence/non adherence, patient provider relation.
3. Discussion may include the strengths of the study and limitations of any implications for the program.

Is the work clearly and accurately presented and does it cite the current literature?

Yes

If applicable, is the statistical analysis and its interpretation appropriate?

Yes

Are all the source data underlying the results available to ensure full reproducibility?

Yes

Is the study design appropriate and is the work technically sound?

Yes

Are the conclusions drawn adequately supported by the results?

Yes

Are sufficient details of methods and analysis provided to allow replication by others?

Yes

Reviewer Expertise:

Operational Research, Tuberculosis, Geriatric health, Epidemiology, Public Health

I confirm that I have read this submission and believe that I have an appropriate level of expertise to confirm that it is of an acceptable scientific standard, however I have significant reservations, as outlined above.

# Response

**Published:** 2022-06-07

**Authors:** Rohith Motappa

**Affiliations:** Kasturba Medical College Hospital, India

Thank you for taking your valuable time for reviewing this article. I have incorporated the following changes in the manuscript based on your suggestions.

 1- The sampling method was followed to select the sites and selection of study participants.

  Response-   With the ongoing pandemic of covid-19, convenient sampling was done to select the multiple tuberculosis units in Mangalore. All the TB patients registered in those Tuberculosis Units were included in the study.  TB cards were used to approach those patients.  The TB patients were approached at their homes/DOTS centers/PHCs with the help of senior treatment supervisors.

 2- Operational definitions followed for certain important terminologies such as adherence/non-adherence, and patient-provider relation.

 Response- 

**Adherence -** Patients who not had missed even a single prescribed dose of TB drug were deemed as Adherent

**Non–Adherence -** Patients who had missed at least one prescribed dose of TB drug were deemed as Non-Adherence

**Patient-Provider Relationship-**  For differentiating between Good Patient-Provider relationships and Poor Patient-Provider relationships, we used 3 key traits of Health workers toward the patients, namely: 

- Empathy towards the patients
- Effective communication with the patients and
- Shared decision-making regarding testing, treatment, and follow-up.

If all the 3 traits were deemed as satisfied by patients, they were categorized as Good Patient-Provider relationships, and even if one trait were not up to the mark according to the patients, it was categorized as Poor Patient-Provider relationships

 3- Discussion may include the strengths of the study and limitations of any implications for the program.

 Response - 

**Strengths of the study**

- This study was done during the Covid-19 pandemic when all the resources and logistics were deviated towards curbing Covid-19 infection. With significant numbers indicating the non-adherence in our study, these study findings showcase that no matter which pandemic might arise, Tuberculosis cannot be brushed aside
- With all the States and UTs working hard toward eliminating TB by 2025, these study findings can provide an insight as to where we stand and how we can improve.

**Limitations of the study **

- As this study was done in Mangalore, these findings cannot be generalized for the Pan-India assumption
- This study considered the TB patients on the whole, without taking any details about the resistance patterns. Prevalence of non-adherence would have been even more comprehensible if we had taken the history of resistance towards any of the anti-tubercular drugs.
